# The power of progressive active learning in floorplan images for energy assessment

**DOI:** 10.1038/s41598-023-42276-x

**Published:** 2023-09-27

**Authors:** Dhoyazan Al-Turki, Marios Kyriakou, Shadi Basurra, Mohamed Medhat Gaber, Mohammed M. Abdelsamea

**Affiliations:** 1 School of Computing and Digital Technology, Birmingham City University, Birmingham, B4 7BD UK; 2 Department of Computer Science, Faculty of Computers and Information, Assiut University, Assiut, 71515 Egypt

**Keywords:** Renewable energy, Environmental impact, Computational science, Scientific data, Software

## Abstract

Floorplan energy assessments present a highly efficient method for evaluating the energy efficiency of residential properties without requiring physical presence. By employing computer modelling, an accurate determination of the building’s heat loss or gain can be achieved, enabling planners and homeowners to devise energy-efficient renovation or redevelopment plans. However, the creation of an AI model for floorplan element detection necessitates the manual annotation of a substantial collection of floorplans, which poses a daunting task. This paper introduces a novel active learning model designed to detect and annotate the primary elements within floorplan images, aiming to assist energy assessors in automating the analysis of such images–an inherently challenging problem due to the time-intensive nature of the annotation process. Our active learning approach initially trained on a set of 500 annotated images and progressively learned from a larger dataset comprising 4500 unlabelled images. This iterative process resulted in mean average precision score of 0.833, precision score of 0.972, and recall score of 0.950. We make our dataset publicly available under a Creative Commons license.

## Introduction

The United Kingdom government has identified approximately 6 million solid wall houses that exhibit inefficiencies and require improvements to enhance their energy efficiency. To tackle this formidable challenge, the government has implemented several initiatives, including the Green Deal Home Improvement Fund (GDHIF), Birmingham Energy Savers, and the Green Deal. These programs have been devised to facilitate the retrofitting of residential properties and other structures, with the goal of achieving energy efficiency enhancements for 60,000 homes. Typically, retrofit projects commence with comprehensive assessments aimed at determining potential energy savings and cost recovery. Given the scale of these projects, non-professionals are involved in conducting these assessments. Presently, energy assessors employ the Standard Assessment Procedure (SAP) and the Reduced Data Standard Assessment Procedure (RdSAP) as recommended by the government^1^. However, it is worth noting that SAP and RdSAP possess certain limitations. Specifically, these methodologies are based on steady-state models that do not account for dynamic heat transfer over time. Furthermore, SAP relies on standardised assumptions, which can potentially yield inaccurate comparisons and advice. In contrast, building energy simulation^2^ offers a more comprehensive testing approach compared to SAP. These simulation tools enable the measurement of interactions between thermal zones, the surrounding environment, and occupants. They simulate combined heat and mass transfer, taking into consideration factors such as air movement, window blinds, and building orientation. Nonetheless, non-engineers may encounter challenges in comprehending and utilising these tools, as they require the creation of 3D building models that accurately replicate existing structures. In recent years, the integration of AI into energy-related studies has witnessed a remarkable upswing. This paper, akin to numerous other research endeavors that explore the use of AI to assess the viability of green energy sources, including wind and solar power, makes use of AI solutions to assess the energy efficiency of buildings. The study conducted by Wang et al.^3^ introduces an approach to assess wind energy potential by employing angular-linear modeling techniques. This methodology encompasses a joint probability density function for wind speed and direction, utilising finite mixture statistical distributions. Wind speed is characterised using a two-component, three-parameter Weibull mixture model, while wind direction is depicted through von Mises mixture models. These models collectively elucidate the intricate relationship between wind speed and direction. Similarly, the work proposed by Bamisile et al.^4^ addresses the challenges associated with evaluating solar energy resources conducive to applications of solar technology. The research compares two hybrid models—ANN-CNN and CNN-LSTM-ANN—along with eight distinct AI models—namely CNN, ANN, LSTM, XG Boost, MLR, PLR, DTR, and RFR. These models are evaluated for their predictive capabilities concerning solar irradiance across diverse time intervals and datasets encompassing six African nations.

Computer vision offers a viable solution for the automated detection of objects within digitised 2D floorplan images^5^. Floorplan images are usually created using tools^6, 7^, where annotations are inserted in a certain order to guarantee consistency and accuracy in labelling. A quality assurance (QA) procedure is in place to regulate the accuracy of the labels and the precision of positioning. The QA procedure consists of two rounds, with the annotator performing the first round and a different QA operator performing the second round, respectively, to guarantee that any potential errors have been fixed. An approach for creating unstructured 3D point clouds into 2D floorplans with topological linkages between walls was proposed in^8^. It consists of two steps: 2D CAD floorplan production and 3D reconstruction. For each floor, floor segmentation based on horizontal planes and wall proposals based on vertical planes is applied in the 3D reconstruction portion to identify the walls. The horizontal projection of wall proposals is utilised to detect walls, and the detected wall points are then used to create an Industry Foundation Classes (IFC) file which is used to develop structural parts in the second section, and the 2D floorplan CAD is produced using these data. It can also be used on Input Data File (IDF), which is an ASCII file containing the data describing the building and HVAC system to be simulated using EnergyPlus^9^. A system for identifying and rebuilding floorplans was proposed in^10^, which is composed of two components: reconstruction and recognition. The floorplan area, structural features, supplemental text and symbols, and scale information from picture pixels are all recognised by the recognition component. The information acquired is transformed into a vectorised expression in the reconstruction phase. The scientists precisely extracted horizontal, vertical, and inclined walls from floorplan photos using a vectorisation technique based on iterative optimisation. Vectorised floor designs were created using two different approaches to depict the walls. One method involves drawing the wall using the edge line, which is a portion of the optimal room contour polygon, while the other involves drawing the wall using the centre line. An image analysis pipeline has been developed in^10^ for floorplan analysis that makes use of a network with outputs for two segmentation maps and a collection of heatmaps. The location and dimensions of all potential elements were inferred from the points of interest located. Finally, the semantics of the floorplan, including the types of rooms and icons, are acquired using the two segmentation maps. In^11^, a floorplan image analysis method based on a four-module strategy was proposed. First, a CNN encoder uses an input floorplan image to extract features. The second module was a room boundary decoder (RBD), to forecast room boundaries such as walls, doors, and windows using the extracted features. Room Type Decoder (RTD) was the third module to predict room types such as the living room, bedroom, bathroom, and closet. Finally, a boundary attention aggregated model (BAAM) unit was employed to make use of feature maps from the RBD and RTD modules as inputs to produce features in order to identify the type of room.

Among the existing floorplan image datasets, the CVC-FP dataset was introduced in^12^, which is annotated for architectural objects and their structural relations. In^12^, a tool has been developed for general-purpose ground truthing. The output of the tool was in standard Scalable Vector Graphics (SVG). The output has mainly focused on wall segmentation and room detection tasks, and a performance evaluation was performed on wall segmentation using the Jaccard Index (JI). ROBIN (Repository Of BuildIng plaNs) is another floorplan dataset that was introduced in^13^, where a deep learning framework, called Deep Architecture for fiNdIng alikE layouts (DANIEL), was proposed to retrieve similar floorplan layouts from a repository. In this approach, the authors perform an extensive analysis comparing the performance of individual hidden layers for the proposed floorplan retrieval task. Several deep object detection models have previously been proposed to deal with objects in floorplan images^14–16^. For instance^7^, utilised an image dataset called CubiCasa5K consisting of 5000 images that have been categorised into more than 80 types of floorplan objects. In^7^, an enhanced multitask convolutional neural network (CNN) was proposed to detect objects in the CubiCasa5K dataset^17^, employed several models, including Mask R-CNN with Deformable Convolutional Networks (DCN)^18^ and Convolutional Neural Networks (CNNs) to deal with the same problem, concluding that DCN outperforms CNNs in detecting objects in 2D floorplan images. In^19^, an instance segmentation model based on Cascade Mask R-CNN was developed, which is supplemented by a new style of key point CNN to accurately extract spatial and geometrical data from floorplan images, resulting in higher segmentation scores compared to existing methods.

This study endeavors to enhance the accessibility of building simulation and optimisation techniques for a wider user base. We introduce and evaluate a novel tool, that has been designed to enable energy assessors to swiftly generate 3D building models from 2D floorplans by leveraging artificial intelligence techniques. The research undertaken was centered around the assessment of energy efficiency in buildings, specifically within the EU-funded project *EcRoFit*. Nevertheless, it is essential to highlight that the outcomes of this study are not confined solely to this project. The work conducted holds a generic nature, enabling its application to diverse building and construction applications. Stating this, the tool proposed in this paper became essential in expediting the energy assessment procedure. Existing intricate tools that provide precise outcomes necessitate the creation of 2D and 3D representations of the property under evaluation, for instance, tools like IES and DesignBuilder. However, this undertaking demands a significant investment of time and proficiency in drawing. Recognising this as an obstacle, our objective was to automate this process while upholding a remarkable level of precision. Despite these challenges and in light of promising outcomes, the tool can be applied to various objectives, such as incorporating expert systems for retrofitting, automatically generating interior designs, and enhancing the property market through virtual viewing, among others. Presently, our focus lies on creating a universal API that can transform 2D floorplans into 3D models, utilizing typical formats like DXF, XML, OBJ, FBX, STL, SKP, GLTF, GLB, and others. This will facilitate the integration of these models into a wide range of 3D software and rendering applications. Consequently, energy assessors can augment the accuracy and efficiency of energy assessments, thereby ultimately advancing the energy performance of buildings and mitigating carbon emissions. Active learning^20^ addresses the challenge of obtaining labelled data by allowing machine learning algorithms to query users interactively for desired outputs, thereby minimising user intervention while achieving more robust annotation tasks and improving model accuracy. In this paper, we propose a progressive active learning workflow that has been trained on a limited number of meticulously annotated images (500 floorplan images) to iteratively rectify the annotation of 4500 intricate images. This iterative process results in a dataset comprising 5000 accurately annotated floorplan images. The processing and annotation of these images have been performed in a manner that provides a benchmark dataset for energy assessors, facilitating a robust and unbiased solution for energy assessment derived from the 3D models of buildings constructed using the identified and requisite objects from the corresponding floorplan images. This paper presents three significant contributions to the field of energy performance assessment:

1. We have created an annotated dataset of floorplan images that is specifically tailored for energy performance assessment applications. This dataset will be a valuable resource for researchers and practitioners in the field.
2. We have developed a novel progressive active learning model that is designed to improve the accuracy of object detection. This model takes into account the uncertainty of the data and uses it to guide the learning process, resulting in more accurate and reliable results.
3. We have introduced a novel computer vision pipeline that is dedicated to floorplan image analysis. This pipeline is specifically designed to facilitate energy performance assessment and will be a valuable tool for researchers and practitioners in the field. It integrates advanced technologies, which encompass deep learning techniques for precise floorplan detection and subsequent generation of 3D models. Our approach is complemented by the integration of EnergyPlus, a state-of-the-art backend engine renowned for its prowess in energy simulation and optimisation.

## Results and discussion

**Figure 1:**
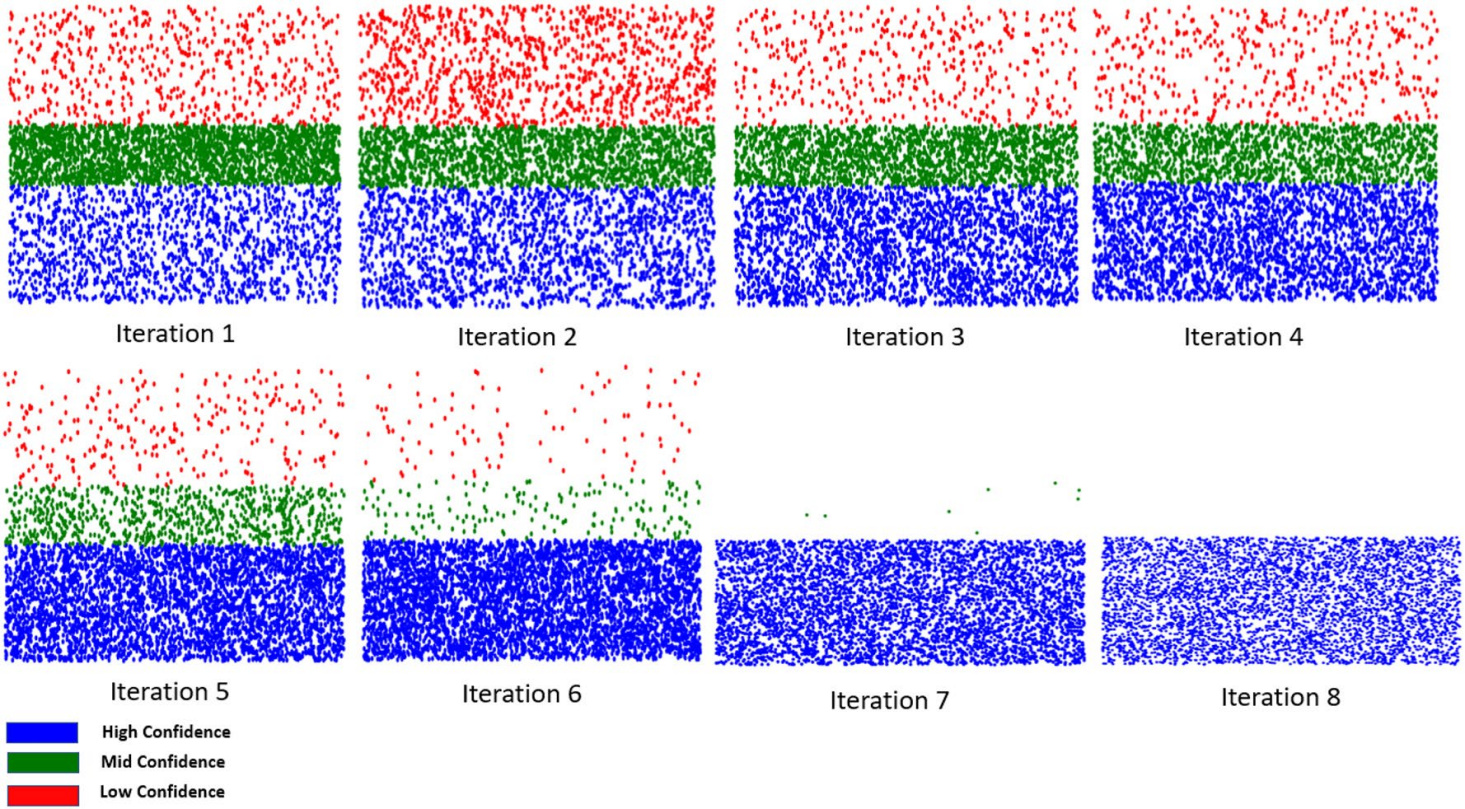
The evolution of the grouping space of the low- mid-, and high- confidence groups during the training process of our active learning model.

This section demonstrates the efficiency and effectiveness of our active learning model in the context of object detection in floorplan images. To evaluate its performance, we initially divided a well-labelled dataset of 500 images into two subsets: a training set (80%) and a test set (20%). The remaining 4500 images were then utilised as input for our active learning model to facilitate annotation. As a result, we obtained a comprehensive dataset consisting of 5000 annotated floorplans. Although the annotations within the dataset are specifically designed to be compatible with YOLO-family models, they can be easily adapted to ensure compatibility with various other object detection models. The evolution of the grouping space by our active learning model is illustrated in Table 1, which shows the gradual adaptation to handle complex samples (see Fig. 1).

**Table 1:** Clusters evolution in terms of the number of samples.

Iteration number	Training samples	High-conf. samples	Mid-conf. samples	Low-conf. samples
1	400	1194	2707	599
2	1594	291	1932	1083
3	1885	796	1789	430
4	2681	246	1526	447
5	2927	943	772	258
6	3870	771	163	96
7	4641	251	8	0
8	4892	8	0	0

### Evaluation metrics

In this work, we adopted the precision, recall, and mean average precision (mAP) matrices to demonstrate the effectiveness of object detection, which are defined as

- Confusion Matrix (*CM*) - A matrix that displays the performance of an algorithm.
- Recall (*R*) - The percentage of real positive values that are correctly identified.
- Precision (*P*) - The percentage of positive identifications that are actually correct.
- Mean Average Precision (*mAP*) - *mAP* is computed by calculating the Average Precision (*AP*) for each class and averaging all results for all objects.
- Intersection over Union (*IoU*) - It measures the overlap between the predicted bounding box and the ground truth bounding box and it is calculated by diving the area of intersection over the area of union.

**Figure 2:**
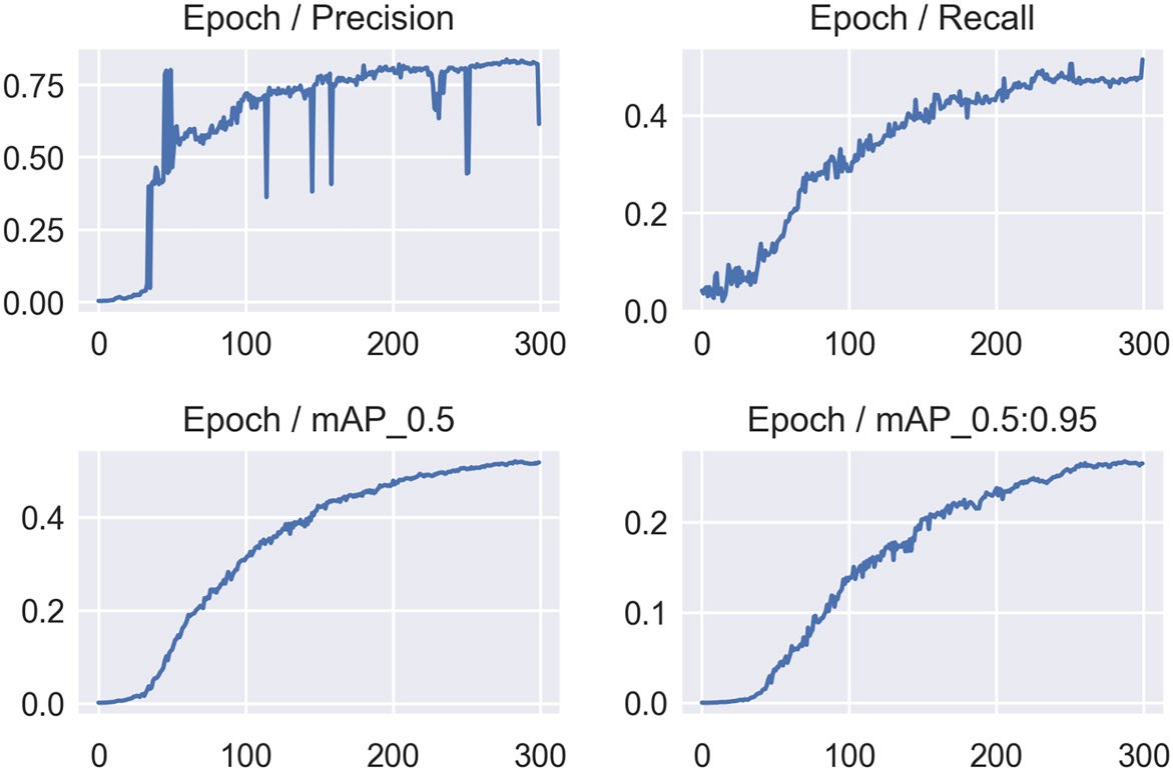
Precision, Recall and mAP with the default hyperparameters, obtained by YOLOv5m on the small annotated dataset.

In our initial experiment, we utilised YOLOv5m^21^ (medium) with a batch size of 32 and 300 epochs and employed the pre-trained COCO^22^ weights. Although the model has a mAp of 0.883, which indicates promising performance throughout the training phase, Figure 2 shows that the model performs noisily in terms of the learning rate.

**Figure 3:**
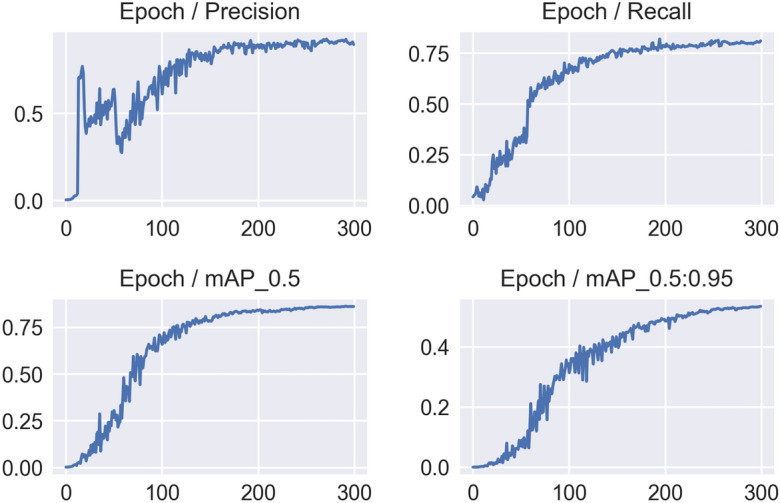
Precision, Recall and mAP with the modified hyperparameter and epochs = 300, obtained by YOLOv5s on the small annotated dataset.

Figure 2 depicts the challenges encountered by the YOLOv5 model in accurately identifying objects within an image, as evidenced by the noise observed in the model’s *mAP*, precision, and recall scores. To address this, we transitioned to training the YOLOv5s (small) model and made a minor adjustment to the initial learning rate (*lr*0), increasing it from 0.01 to 0.001. Given that the pre-trained weights are derived from the COCO dataset, which focuses on real-world objects, we opted to train the YOLOv5s model from scratch in an end-to-end manner, without leveraging the pre-trained COCO weights. Consequently, the YOLOv5s model achieved a slightly lower *mAP* score of 0.833, with precision and recall scores of 0.778 and 0.824, respectively. Despite the slight decrease in *mAP* score resulting from the modified learning rate, the model exhibited improved performance throughout the learning process. Figure 3 illustrates the performance of the newly employed YOLOv5s model over the course of 300 epochs. A sequence of experiments was undertaken using YOLOv5s, encompassing 300 epochs in each instance. Within each experiment, the model’s prediction was informed by the most effective epoch weights obtained during training. Following this prediction phase, a process of iterative refinement was initiated, involving the reannotation of samples. This iterative approach facilitated continual enhancements in the training process, with the model being retrained to accommodate the refined annotations. This iterative refinement process persisted until the training culminated with annotations derived from high-confidence samples.

**Figure 4:**
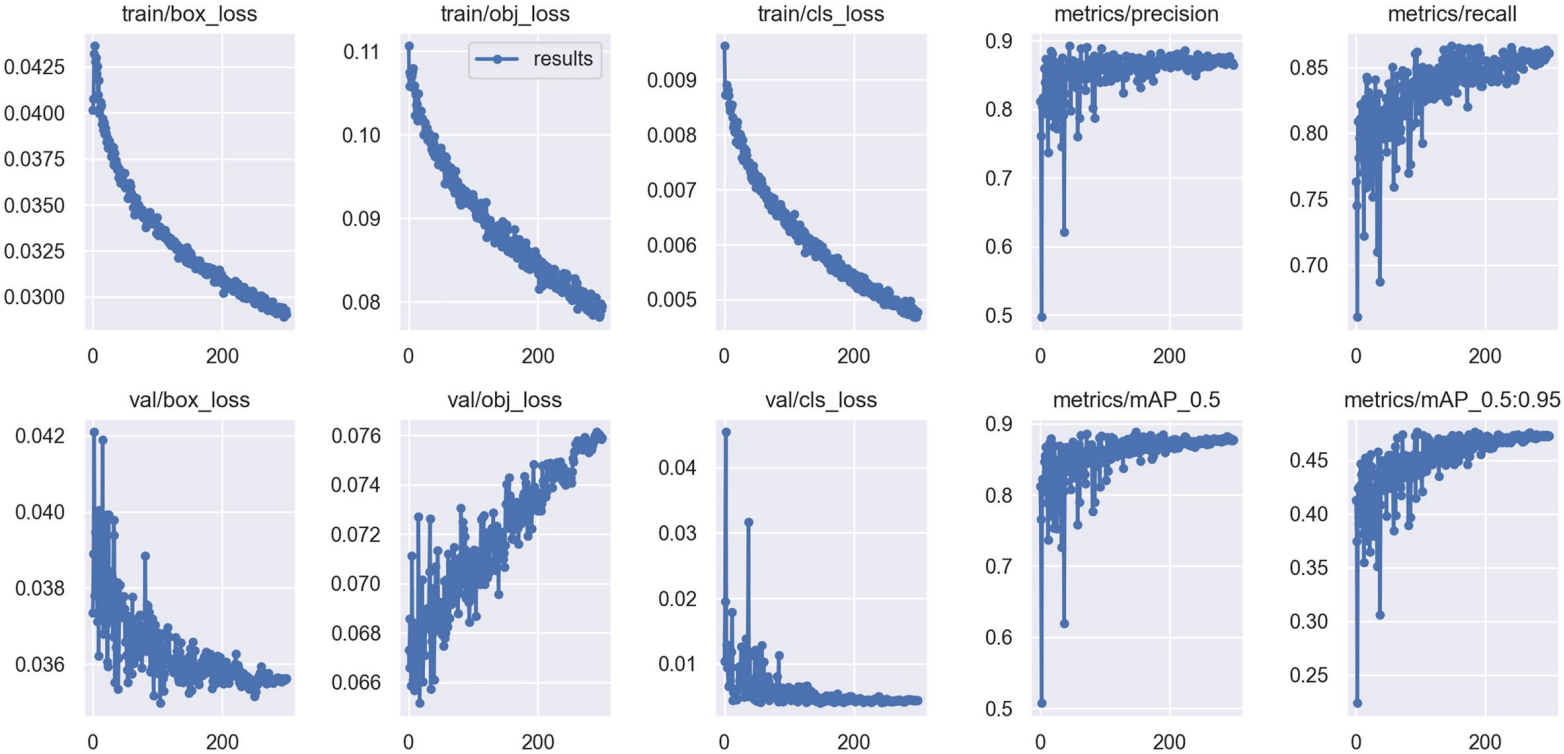
The results of different metrics obtained by YOLOv5 after 300 epochs on the complete dataset.

**Figure 5:**
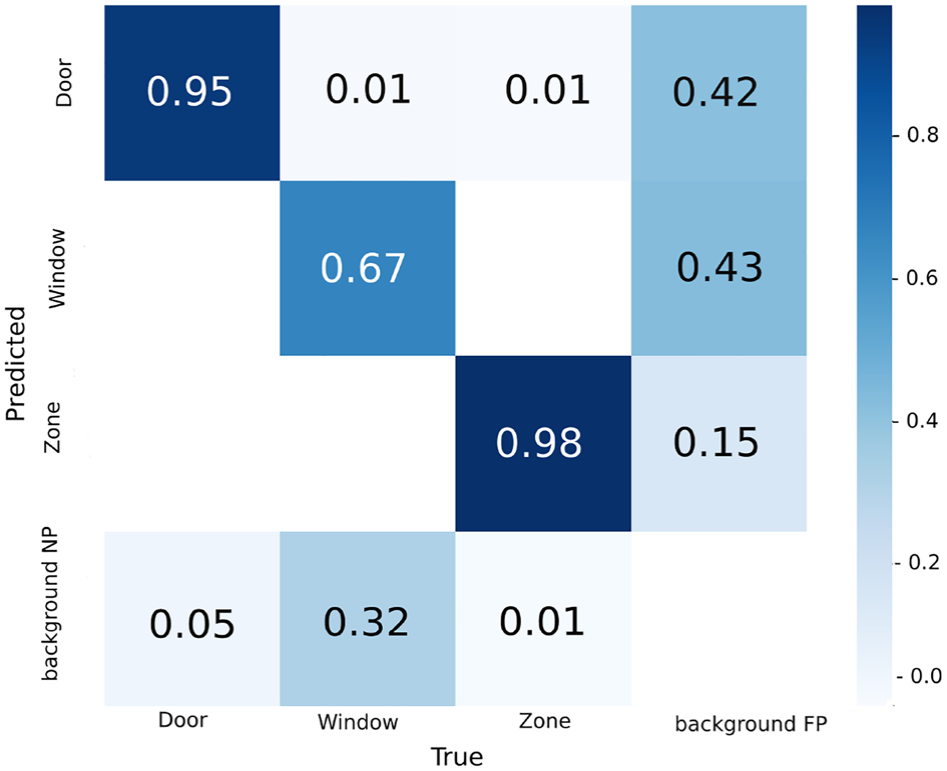
The confusion matrix of the training set with 300 epochs.

Figures 4 and 5 demonstrate the overall performance of YOLOv5s on the given dataset in terms of the main evaluation metrics.

**Figure 6:**
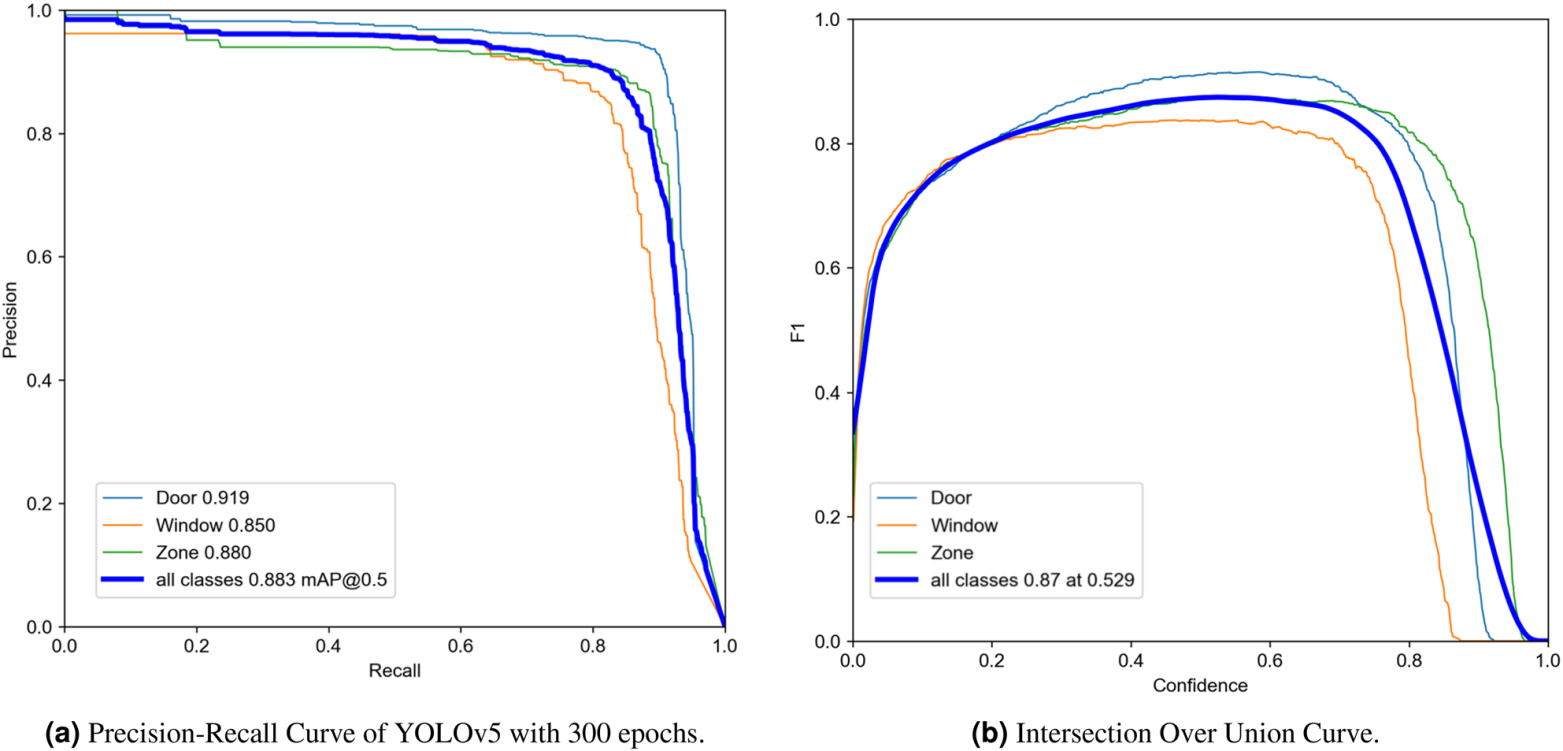
Precision–Recall and intersection over union.

Figure 6a presents the precision-recall curve, which illustrates the trade-off between precision and recall at various thresholds (where a larger area under the curve indicates higher precision and recall), and Figure 6b shows the intersection over union curve (IOU). Additionally, accuracy is inversely related to the false positive rate, while recall is inversely related to the false negative rate. Our model exhibits a precision-recall pattern that bows towards the point (1, 1), indicating a well-performing model. The combined precision-recall scores for the door, room, and window classes were 0.841, 0.869, and 0.763, respectively, resulting in an overall score of 0.824 for our model.

## Conclusion

This paper introduces an active learning workflow designed to detect three key elements (zones, windows, and doors) within floorplan images, aiming to facilitate automated and efficient energy assessment tasks for energy assessors. The active learning model was trained using a limited number of meticulously annotated images to iteratively improve the annotation quality of 4500 challenging images. The resulting dataset has been meticulously processed and annotated, serving as a benchmark for achieving a robust and unbiased solution for energy assessment. This work will help in assessing energy performance, encompassing construction, installation, and building services that prioritise energy efficiency.

## Materials and methods

### Dataset

In this section, we provide a comprehensive discussion of the dataset used in this study, including its original sources and a brief comparison with other existing datasets, as presented in Table 2. The following datasets are commonly utilised for floorplan image analysis:

#### ROBIN (Repository Of buildIng plaNs)

ROBIN^13^ consists of 510 floorplans that are used to automatically find existing building layouts from a repository that may help the architect ensure reuse of the design and timely completion of projects. They propose Deep Architecture for fiNdIng alikE Layouts (DANIEL), so an architect can search from the existing project’s repository of layouts (e.g., floorplan) and give accurate recommendations to the buyers.

#### CVC-FP: database for structural floorplan analysis

CVC-FP^12^ has 122 floorplans that are annotated for architectural objects and their structural relations. They have implemented a ground truthing tool, named the SGT tool, which allows us to make this kind of information specific in a natural manner. The tool allows one to define own object classes and properties, multiple labeling options are possible, and grants cooperative work. The dataset is fully annotated for structural symbols: rooms, walls, doors, windows, parking doors, and room separations.

#### CubiCasa5K: a dataset and an improved multi-task model for floorplan image analysis

CubiCasa5K^7^ is a large-scale floorplan image dataset containing 5000 samples annotated of over 80 floorplan object categories. The annotations of the dataset are performed in a dense and versatile manner using polygons to separate the different objects.

**Table 2:** A comparison between existing floorplan image datasets.

Name	Size	Purpose
ROBIN	512 images	Deep learning approaches to automatically analyse building floorplan images and retrieve similar plans from a large-scale repository. The proposed technique can find application in an online property sale/rent scenario where the buyer has preferred features related to the room semantics.
CVC-FP	122 images	The dataset is fully ground-truthed for the structural symbols: rooms, walls, doors, windows, parking doors, and room separations. It not only makes their locations in the images specific but also includes structural relations between them that are of special interest for analysis systems.
CubiCasa5K	5000 images	Multi-task learning scheme which uses the ’multi-task uncertainty loss’ based on 5000 images and starts detecting 80 different element classes inside the floorplan.

#### Our dataset

Our dataset comprises 5000 images obtained from the aforementioned open-source datasets. Initially, 500 images were manually annotated, and subsequently, we propose the utilisation of an active learning model to aid in annotating the remaining images. The active learning model focuses on detecting three distinct classes, namely rooms, windows, and doors, ultimately enabling the generation of a 3D model of the building. This work is part of a proposed framework for assessing energy performance, which encompasses construction, installation, and building services dedicated to enhancing energy efficiency. It specifically caters to property energy professionals and retrofit professionals. It is worth noting that the combined total of samples from the three data sources amounts to 5634. However 5000 samples were selected in a way to avoid duplication as follows:

- 300 samples from ROBIN dataset
- 100 samples from CVC-FP dataset
- 4600 samples from CubiCasa5K dataset

Then, we selected 500 images from these 5000 images randomly for manual annotation.

### Methodology

**Figure 7:**
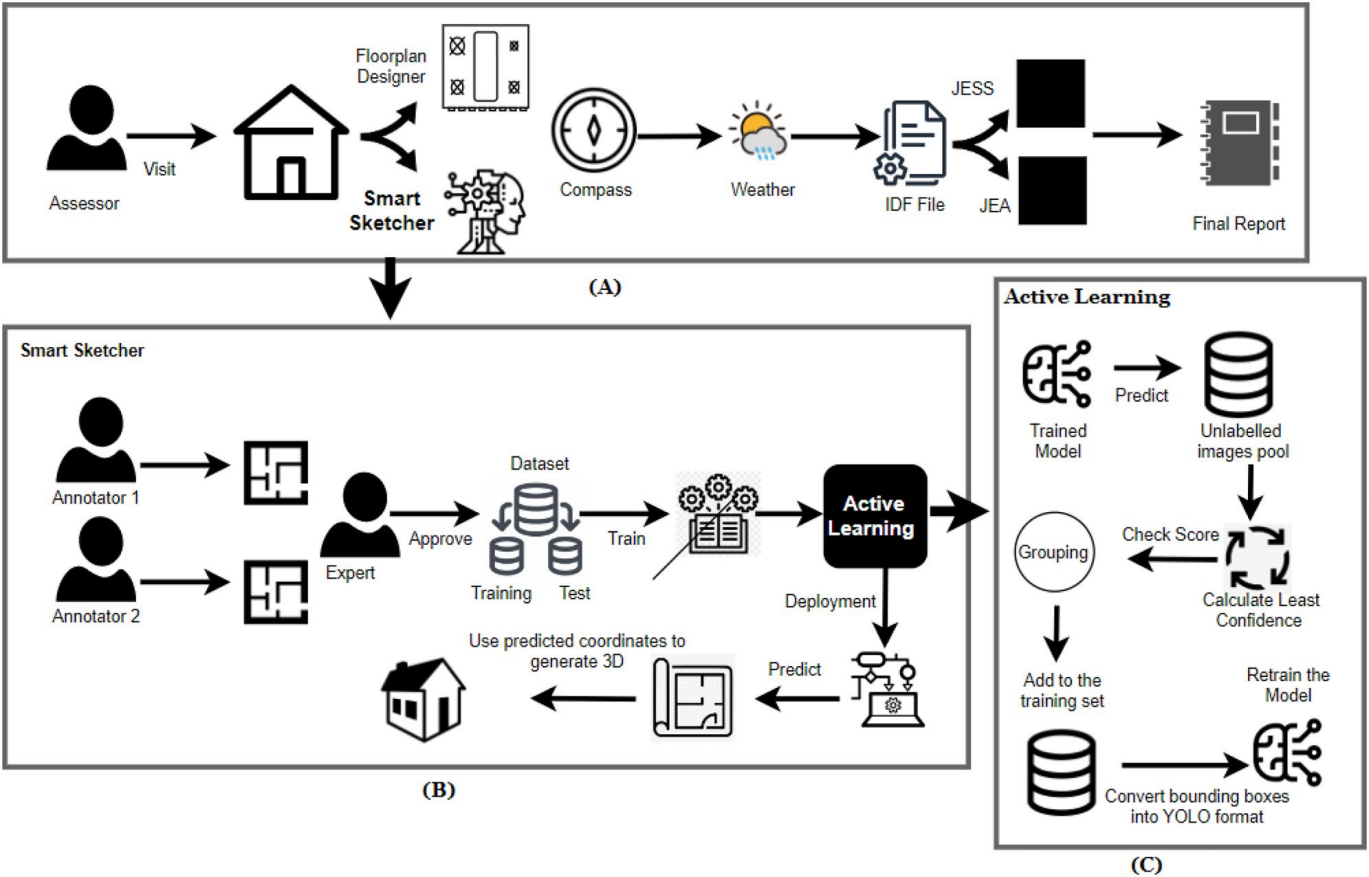
The generic architecture and main components of our proposed pipeline for energy simulation and optimisation (**A**), the annotation process and Smart Sketcher (**B**) and the proposed Active Learning technique (**C**).

Our proposed pipeline has been designed in a way to offer an easy-to-use, highly visual interface to create fast and accurate energy assessments, see Fig. 7. It applies multi-objective optimisation techniques to compare multiple options for holistic retrofit measures and can compare and contrast variations of product specifications. The main components of our proposed pipeline can be described as

- Floor designer: This component is used by the performance energy assessor to draw rooms, windows, doors, and floors of a house and add some materials and constructions for a building to be used for the performance energy assessment. The user will start creating a floor, then define zones (rooms) within this floor, then add windows and doors for each zone (room), then add the construction materials used for all walls (internal and external), windows and doors, and repeat this process for each floor of this house to finally define the type of roof for this house and its construction material.
- Smart sketcher: This is a machine learning model that takes floorplan images as input, detecting rooms, windows, and doors, and then converting 2D floorplans into 3D models through four steps: element detection, external framing with OpenCV, 3D model generation using ThreeJS, and external view creation. It interacts via Flask web API or can be integrated into applications using Google TensorFlow Lite Converter.
- Light compass: This component is used to define the house orientation (North, South, East, or West), location, and the weather data to be used as parameters for a performance energy assessment. The user will start defining the orientation with the help of the mobile/tablet built-in orientation sensor, then use the device location sensor to define the house location and city (where based on the city, the user can call a web API to retrieve the weather data for this location).
- All the collected data by the assessor will be stored inside .idf (Input Data File) which is the required format for the EnergyPlus engine. This includes many parameters such as HVAC system, construction and materials used for the building, orientation and many more. IDF is an ASCII file that contains the data that describe the building and HVAC system to be simulated. The data is stored as key / value paired as shown below: Material:NoMass, R19LAYER, !- Name Rough, !- Roughness 3.344,                     !- Thermal Resistance 0.9000000,                !- Thermal Absorptance 0.7500000,                !- Solar Absorptance 0.7500000;                !- Visible Absorptance
- JESS (JEplus Simulation Service): they are a set of web APIs that are used for the online simulation service for EnergyPlus. It receives the .idf file generated from the above components and retrieves a report showing the predicted gas consumption, electricity consumption, and CO2 emissions for the building.
- JEA: is a parametric, sensitivity analysis, and optimisation engine used to generate different results of energy performance based on different situations and then recommend the best option for the building.
- Flask web API is a gateway between the trained AI models and the web version of the solution.
- Tensorflow Lite is a light version of the machine learning model developed to support the mobile version of the trained models.

The pipeline will generate a comprehensive report presenting a multitude of results, including but not limited to heating, cooling, electricity and gas consumption, CO2 emissions, and numerous other metrics.

#### Floorplan annotation

**Figure 8:**
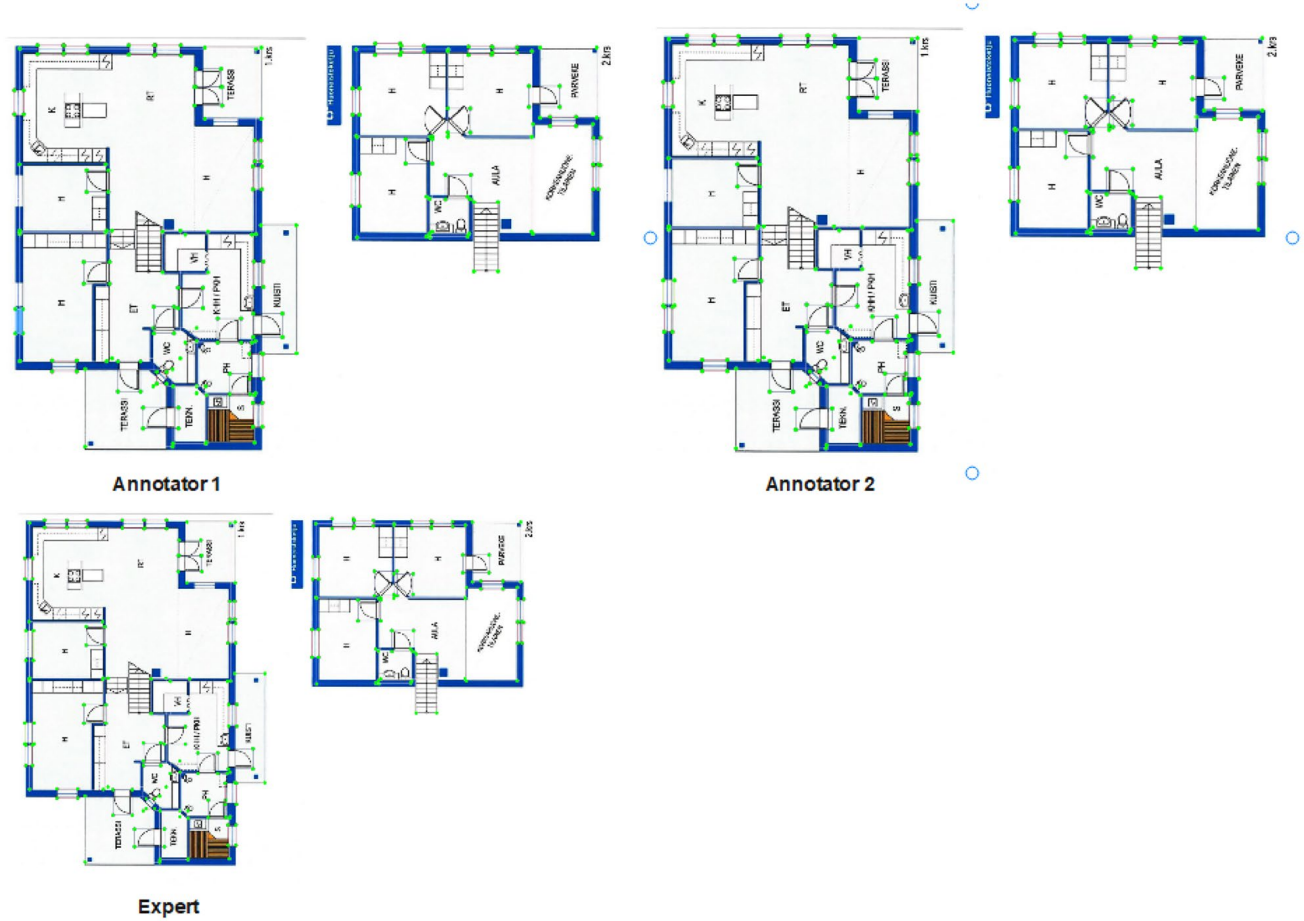
The first annotator missed 1 door and 2 windows, the second annotator 2 missed 1 window, the last one annotated by the expert.

In this study, the bounding box technique was employed to annotate our image dataset, offering compatibility with a wide range of object detection deep learning models. To ensure annotation consistency and accuracy, we enlisted the expertise of two independent annotators who manually delineated visual boxes around the objects of interest within each image. The resulting annotations, comprising the class ID, coordinates (x, y) representing the center of the boundary, and the dimensions of the box (length and width), were saved in a .txt file format. Following the annotation process, annotations with the highest level of agreement between the two annotators were selected, ensuring reliability and inter-rater consistency. Additionally, a third expert annotator meticulously reviewed all annotated objects, making necessary corrections for any mislabeled instances. This rigorous process yielded precise annotations for the initial dataset, comprising 500 images, which served as a prerequisite for our active learning model to complete the annotations for the entire dataset (see Fig. 8).

#### Object detection and active learning model

**Figure 9:**
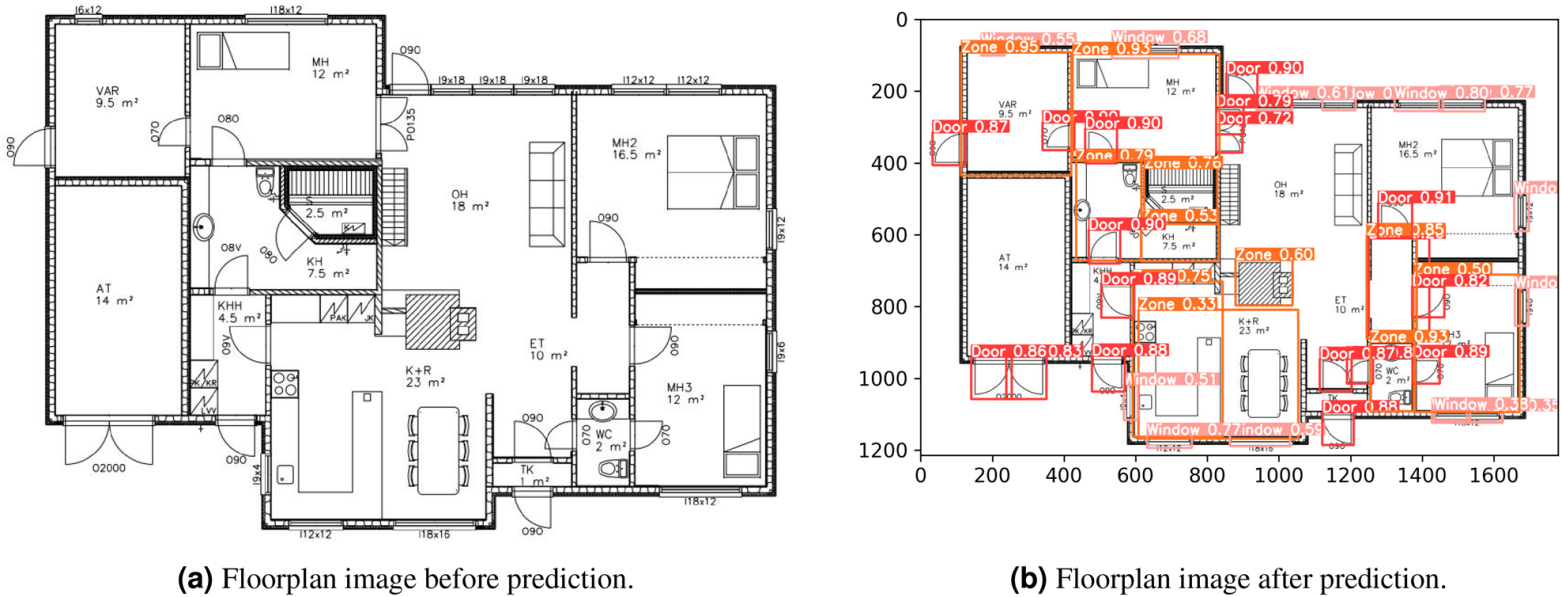
Floorplan before and after prediction using YOLO.

Here we utilised the YOLO-v5 model to train on a carefully annotated dataset of 500 floorplan images. Out of these, 400 images were allocated for training and validation purposes, while the remaining 100 images were reserved for testing the trained YOLO-v5 model. The training process involved 500 epochs with a batch size of 16, following meticulous fine-tuning of hyperparameters. Subsequently, we initiated the training process of our active learning technique using the pool of unlabelled images within the dataset, consisting of 4,500 samples. YOLO-v5 was employed on this initial pool to detect objects and calculate prediction probabilities, see Fig. 9. These probabilities served as an indication of the model’s confidence in detecting and classifying objects in the floorplan images.

Consequently, we utilised these confidence scores to partition the initial pool of floorplan images into clusters based on prediction uncertainty. Specifically, we divided the 4500 images into three distinct groups, namely ‘high-confidence’, ‘middle-confidence’, and ‘low-confidence’.

The ‘high-confidence’ cluster comprises samples associated with high probability values, indicating that the object detection model successfully handles these samples. Conversely, the ‘low-confidence’ cluster encompasses challenging samples where the object detection model struggles to provide accurate predictions. The pool of unlabelled images is dynamically updated throughout the iterative process. Samples within the high-confidence cluster are utilised to retrain the object detection model and subsequently removed from the pool. In each iteration, the grouping space is reevaluated, and the process is repeated until convergence is achieved, wherein no unlabelled samples remain. This iterative approach enables our active learning technique to progressively enhance the model’s performance and actively select the most informative samples for annotation.

In order to construct the cluster space, we introduced a novel metric referred to as the $\beta$-score, which builds upon the Least Confidence (*LC*) metric for evaluating the overall performance on an unlabelled test image. Given that each image encompasses multiple elements such as doors, windows, and rooms, the $\beta$-score was defined as the average of the Least Confidence scores for all bounding boxes within an image. Specifically, we computed the least confidence score for each bounding box in an image and subsequently calculated the average of these least confidence scores for all bounding boxes within the same image. This calculation is represented by Eq. 1:

1 $$\begin{aligned} \beta _{avg}(I) = \frac{ \displaystyle \sum _{i=1}^{n} LC_{i}}{n}, \end{aligned}$$

where *n* is the number of elements in a test unlabelled image *I*(*x*), *x* is the pixel location. $LC_{i}$ is the least confidence score associated with each element *i* in the image *I*, which is defined in Eq. 2.

2 $$\begin{aligned} LC_{i}(I)=1-C, \end{aligned}$$

where *C* is the prediction probability obtained by the trained YOLO-v5 model. $LC_{i}$ are measured for all the bounding boxes in an image and then the average least confidence score ($\beta _{avg}(I())$) is calculated. The pool of unlabelled images is clustered into three groups based on the confidence scores of the images (high, medium and low), where we used a trial-and-error method to achieve cluster assignment.

Our proposed grouping method for floorplan images was designed to enhance the efficiency and effectiveness of our active learning model. The iterative update of the cluster space aims to maximise the number of samples in the high-confidence cluster while minimising the number of samples in the other clusters. Through this approach, our active learning model is retrained iteratively using new samples from the high-confidence cluster, while simultaneously updating the grouping space. As a result, our active learning model progressively filters out challenging samples by transferring them from the low/mid-confidence clusters to the high-confidence cluster, which are subsequently included in the training set for retraining YOLO-v5. This gradual transition enables YOLO-v5 to acquire the ability to handle difficult samples, ultimately relocating all samples from the low/mid-confidence clusters. Furthermore, to enhance the model’s robustness, we have incorporated an expert intervention component that reviews and corrects predictions before incorporating samples into the training set.

In algorithm 1, we describe the implementation flow of our progressive active learning model, see also Fig. 7.
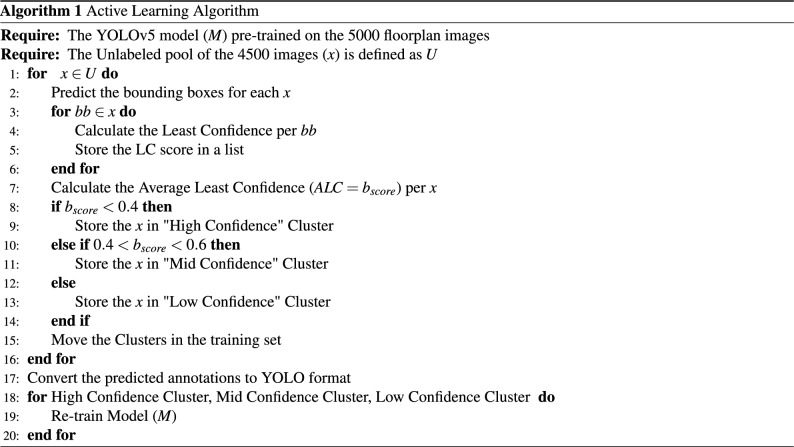


#### Generating 3D model

After obtaining the coordinates of the bounding boxes for the three categories (zones, windows, and doors), we processed the zone coordinates by converting them into three-dimensional cubes. This was achieved by adding a fixed height to the zone coordinates, as illustrated in Algorithm 2.
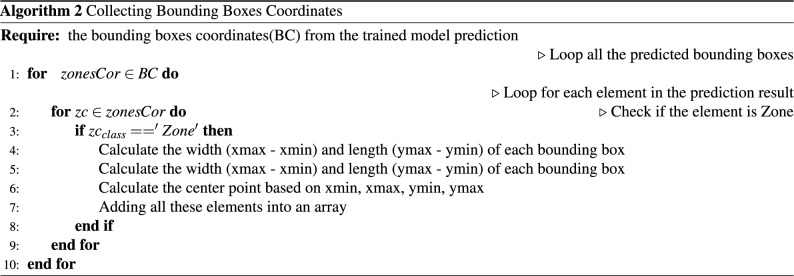


**Figure 10:**
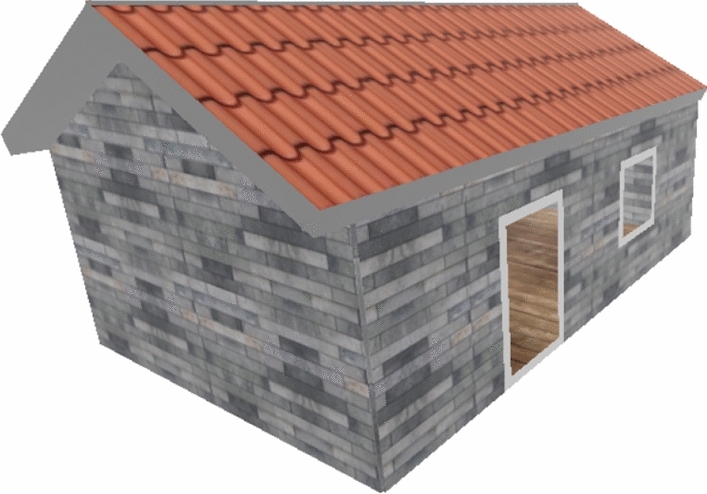
3D Model generated using the detected objects from a 2D floorplan image and ThreeJS library.

For energy performance assessment, the detection of windows and external doors was accomplished using the OpenCV library, supplemented by manual height adjustments representing 75% of the zone’s height. The visual output was then realized through ThreeJS, a cross-browser JavaScript library utilizing WebGL to create and display animated 3D graphics within web pages, see Fig. 10.

### Ethical approval

We confirm that the experimental study is in accordance with relevant institutional, national, and international guidelines and legislation.

## Data Availability

We have released the dataset under a Creative Commons Attribution 4.0 International License, which can be downloaded at https://sandbox.zenodo.org/record/1206021.
